# Vaccination of Mice with a Novel Trypsin from *Trichinella spiralis* Elicits the Immune Protection against Larval Challenge

**DOI:** 10.3390/vaccines8030437

**Published:** 2020-08-05

**Authors:** Yao Zhang, Jie Zeng, Yan Yan Song, Shao Rong Long, Ruo Dan Liu, Peng Jiang, Xi Zhang, Jing Cui, Zhong Quan Wang

**Affiliations:** Department of Parasitology, Medical College, Zhengzhou University, Zhengzhou 450052, China; zhangyao0815@gs.zzu.edu.cn (Y.Z.); zengjie950316@gs.zzu.edu.cn (J.Z.); songyanyan2020@gs.zzu.edu.cn (Y.Y.S.); srlong@zzu.edu.cn (S.R.L.); liuruodan@zzu.edu.cn (R.D.L.); jiangp@zzu.edu.cn (P.J.); zhangxi@zzu.edu.cn (X.Z.); cuij@zzu.edu.cn (J.C.)

**Keywords:** *Trichinella spiralis*, foodborne parasite, trypsin, immune protection, ADCC

## Abstract

*Trichinella spiralis* is a major foodborne parasite and has a serious threat to meat safety. Development of anti-*Trichinella* vaccines is prospective to eliminate *Trichinella* infection in food animal. The aim of this study was to assess the biological properties of a novel *T. spiralis* trypsin (TsT) and its elicited immune protection against larval challenge. The cDNA sequence of TsT gene was cloned and expressed. Western blotting showed rTsT was identified by infection serum and anti-TsT serum. RT-PCR results revealed that TsT gene was transcribed at diverse *T. spiralis* lifecycle stages. The IIFT results showed that natural TsT was principally expressed at epicuticle of 5-6 day adult worms, indicating that TsT is a worm somatic antigen and adult-stage specific surface antigen. Vaccination of mice with rTsT triggered an evident humoral immune response (high levels of serum IgG, IgG1/IgG2a, and enteral sIgA), and it also induced the systemic and enteral local cellular immune response, demonstrated by an significantly elevation of cytokines IFN-γ and IL-4. The mice vaccinated with rTsT exhibited a 33.17% reduction of enteral adult worms and a 37.80% reduction of muscle larvae after larval challenge. The results showed that TsT might be considered as a candidate target antigen for anti-*T. spiralis* vaccines.

## 1. Introduction

*Trichinella spiralis* is a major zoonotic parasitic nematode with a worldwide distribution in more than 150 kinds of mammal animals [1]. Human *Trichinella spiralis* infection is primarily resulted by the consumption of raw or semi-cooked animal meat infected with encapsulated infective larvae. According to a global risk-ranking conducted by FAO/WHO, *T. spiralis* was ranked the greatest importance in meat [2]. The domestic pig pork is the dominant infection source of human *Trichinella* infection in China and other developing countries [3,4]. Fourteen trichinellosis outbreaks owing to pork and wild boar meat were reported in the mainland China [5]. Since a mass of domestic pork was consumed over the world, swine *T. spiralis* infection is a severe threat to the meat safety and public health [6,7]. Therefore, it is needed to explore anti-*Trichinella* vaccine to interdict *Trichinella* infection in pigs and eradicate the infective larvae in animal food [8,9].

After being ingested, encapsulated *T. spiralis* muscle larvae (ML) in muscle tissues are released from the collagen capsules with the aid of digestive fluid, develop to intestine infective larvae (IIL) after being contacted with enteral contents or bile [10,11]. The IIL intrude into enteral mucosal epithelium and develop to adult worms (AWs) after they molt four times. Following copulation, the pregnant adult females generate newborn larvae (NBL) which enter the blood circulation, invade into skeletal muscles and become the encapsulated larvae to complete the lifecycle [12]. The enteral epithelium is the first natural defense barrier against *T. spiralis* invasion, and the major interaction location of host and the parasitic nematode [13,14], but the mechanism of enteral epithelium invasion by *T. spiralis* larvae is unclear [15,16].

Trypsin is a main subfamily of serine protease superfamily in helminths [17]. Serine proteases play a major biological funcion during *Trichinella* infection, and they are involved in parasite invasion, migration and degration of host’s various tissue components [18,19]. In recent years, some kinds of serine proteases were identified in the excretion/secretion (ES) and surface proteins of different *T. spiralis* stages by proteomics/immunoproteomics [20,21,22,23,24]. While *T. spiralis* IIL larvae were inoculated on the monolayer of intestinal epithelium cells (IECs) and co-cultured, the IIL larvae penetrated into the IEC monolayer and produced additional serine proteases which passed into IECs [25,26]. *T. spiralis* serine proteases are likely to promote the larval invasion of enteral mucosa and enteral *T. spiralis* infection [27,28]. However, immunization of mice with some kinds of single recombinant serine protease only exhibited a partial protection against *T. spiralis* challenge [29,30,31,32]. Therefore, it is needed to identify and characterize additional novel serine proteases from *T. spiralis* and evaluate their elicited immune protection.

In the present study, a novel *T. spiralis* trypsin gene (TsT, GenBank: XP_003374437.1) was retrieved from *T. spiralis* draft genome [33]. The aim of this study was to ascertain the biological properties of TsT during *T. spiralis* infection and the immune protection induced by vaccination with the rTsT in a model of BALB/c mice.

## 2. Materials and Methods 

### 2.1. Parasite, Animal and Antigens

*Trichinella spiralis* strain (ISS534) was collected from a domestic pig in central China and passaged in BALB/c mice in our laboratory [34]. Female BALB/c mice (4–6 week-old) were gotten from Henan Experimental Animal Center. The ML were obtained via artificially digestion of infected murine carcasses 42 days post infection (dpi) as reported before [35]. The IIL and AW were recovered from small intestine of infected mice at 6 hpi, 3, 5 and 6 dpi [36]. Female adults at 5 dpi were cultivated in RPMI-1640 with 10% fetal bovine serum (FBS; Gibco) at 37 °C for 24 h, and the NBL were harvested [37,38]. The ML soluble somatic proteins and ES proteins were prepared as previously described [17,39].

### 2.2. Bioinformatics Analysis of TsT Gene 

The full-length TsT cDNA sequences were retrieved from GenBank (No: XP_003374437.1). The physicochemical properties of TsT were analyzed by bioanalysis software and websites. The signal peptide and subcellular localization were predicted as described [40]. The amino acid sequence of TsT gene was compared with trypsin from other organisms as reported before [41]. The species and accession numbers of trypsin sequences are as follows: *Trichinella nativa* (OUC47977.1), *Trichinella britovi* (KRY60943.1), *Trichinella pseudospiralis* (KRX95232.1), *Trichinella murrelli* (KRX46163.1), *Trichinella* sp.T6 (KRX79512.1), *Trichinella nelsoni* (KRX19144.1), *Trichinella* sp.T8 (KRZ86511.1), *Trichinella* sp. T9 (KRX60603.1), *Trichinella papuae* (KRZ70120.1), *Trichinella zimbabwensis* (KRZ14485.1), *Trichinella patagoniensis* (KRY19647.1), *Trichuris trichiura* (CDW57390.1), *Trichuris suis* (KFD53607.1), *Mus musculus* (EDL22287.1), and *Homo sapiens* (EAX06317.1). Phylogenetic tree was constructed with corresponding sequences of 14 species by using neighbor-joining (NJ) method in MEGA 7.0 (http://www.kumarlab.net) [42].

### 2.3. Cloning, Expression and Identification of TsT

Total RNAs were isolated from the muscle larvae with Trizol (Invitrogen, Carlsbad, CA, USA). The TsT cDNA sequences without signal peptide were amplified using PCR with primers carrying *Bam*HI and *Hind*III restriction sites (**bold**) (5′-AAAAA**GGATCC**GCCAAAAAGGCCACCACCAACTA-3′; 5′-CCCGC**AAGCTT**TTAAGCCACTCTAATAGAGTTC-3′). The PCR products were sub-cloned into the pQE-80L expression vector and recombinant vector containing TsT gene was transformed into *Escherichia coli* BL21 (DE3) (Novagen, La Jolla, CA, USA). Expression of rTsT protein was induced using 0.5 mM IPTG for 5 h at 37 °C [15], and purified with Ni-NTA-Sefinose resin (Sangon Biotech Co., Shanghai, China) [43,44]. The concentration of rTsT was measured and analyzed on SDS-PAGE and Western blotting as previously reported [45,46]. 

### 2.4. Immunization of Mice and ELISA Determination of Anti-rTsT Antibodies

**Figure 1:**
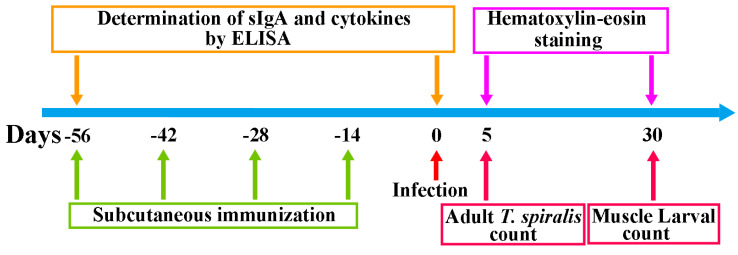
Scheme of immunization and measure protocol. Subcutaneously immunization of mice was administered four times (days-56, -42, -28 and -14) at a 2-week interval before orally challenged with 300 *T. spiralis* ML on day 0. At day 5 and 30 after challenge, intestinal adult worm and muscle larval burden (larvae per gram, LPG) were respectively assessed to evaluate the immune protection induced by the rTsT immunization. Histopathological examination of muscle and intestines from infected mice was also performed at day 5 and 30 after larval challenge infection.

Ninety mice were randomly divided into 3 groups of 30 mice each. The rTsT immunizaton group was subcutaneously vaccinated with 20 µg rTsT emulsified with the adjuvant ISA 201 (Seppic, Paris, France), and boosted three times at a 2-weeks interval using rTsT with ISA 201. Adjuvant and PBS control groups received only ISA 201 or PBS at the same time points as experimental group [47]. About 100 μL tail blood were obtained from each mouse at weeks 0, 2, 4, 6 and 8 after vaccination, and sera were isolated and stored at −80 °C until use [48]. The scheme of immunization and measure protocol was shown in Figure 1.

Specific anti-rTsT antibody IgG, and IgG1/IgG2a in all vaccinated mice were assayed using routine ELISA with rTsT protein [49]. In brief, the ELISA plate was coated with 2 μg/mL rTsT at 37 °C for 2 h. After washes using PBST, the plate was blocked with 5% skimmed milk at 37 °C for 2 h. After washing again, the plates were incubated at 37 °C for 2 h with 1:100 dilutions of mouse immune sera, and then with HRP-labelled anti-mouse IgG (IgG1/IgG2a; 1:5000 dilutions; Sigma-Aldrich, St. Louis, MO, USA) for 1 h at 37 °C. The plates were colored with OPD (Sigma-Aldrich) plus H_2_O_2_, the reaction was finished by addition of 2 M H_2_SO_4._ The absorbance (optical density, OD) values at 492 nm were measured by a microplate reader (TECAN, AG, Switzerland) [44,50].

### 2.5. Western Blotting Analysis of rTsT Antigenicity

The ML soluble somatic crude proteins, ES proteins and rTsT were separated on 12% SDS-PAGE. The proteins were transferred onto nitrocellulose membrane (Merck Millipore, Billerica, MA, USA) [51,52]. The membrane was blocked with 5% skimmed milk diluted in TBST at 37 °C for 2 h, and cut into strips. The strips were incubated with different sera (1:100 dilutions of anti-rTsT serum, infection serum and normal serum) at 37 °C for 2 h. After washing with TBST, the strips were incubated with HRP-labelled anti-mouse IgG (1:5000; Southern Biotech, Tuscaloosa, AL, USA) at 37 °C for 1 h. Following washes again, 3, 3′-diaminobenzidine tetrahydrochloride (DAB; Sigma-Aldrich) was used for the detection of positive reaction [53].

### 2.6. RT-PCR Analysis of TsT mRNA Expression in Diverse T. spiralis Stages

Total RNAs from ML, IIL, 3-day AW, 6-day AW and NBL were isolated with Trizol reagent (Invitrogen). RT-PCR was conducted to ascertain the TsT mRNA expression levels in various *T. spiralis* stages as previously described [54]. A housekeeping gene GAPDH from *T. spiralis* (GenBank: AF452239) was also amplified and served as an internal control [55]. PBS was used as a negative control in all PCRs.

### 2.7. Indirect Immunofluorescence Test (IIFT)

Fresh complete worms of various *T. spiralis* stages (ML, IIL, AW and NBL) were fixed in cold acetone for 20 min. Furthermore, the ML, IIL and AW were embedded in paraffin, 3-µm thick cross-sections were prepared using a microtome. Expression and tissue localization of native TsT in various *T. spiralis* stages were determine with IIFT [16,56]. In brief, the whole worms and worm cross-sections were blocked using 1% BSA in PBS and then incubated with various sera (1:10; anti-rTsT serum, infection serum or pre-immune serum) at 37 °C for 2 h. Following washes with PBS, worms and cross-sections were stained with anti-mouse IgG conjugated FITC (1:100; Santa Cruz Biotech, Dallas, Texas, USA). After washes again, the whole worms and cross-sections were examined under a fluorescence microscopy (Olympus, Tokyo, Japan) [57,58].

### 2.8. Assay of Total IgA and TsT-Specific IgA in Enteral Fluid

To detect secretory IgA (sIgA) in enteral secretion, enteral secretions were collected as described [8,59]. In brief, a 20 cm length of complete small intestine (duodenum, jejunum and ileum) was excised and cut into three enteral segment. Cold PBS with 1% protease inhibitor (Sangon Biotech, Shanghai, China) was flushed through the intestine. The washing fluid was harvested. The sample was pooled and centrifuged at 5000× *g* at 4 °C for 5 min, and the supernatant was recovered [29]. Total enteral sIgA was measured with a sandwich ELISA [57]. TsT-specific sIgA was detected using ELISA with 2 μg/mL muscle larval crude antigens or rTsT as described [30]. The plate was colored using OPD and the optical density (OD) at 492 nm was measured with an ELISA reader as described before [60].

### 2.9. Assay of TsT-Specific Cytokines 

To assess the TsT-specific cellular immune responses, five mice of each group were killed before immunization and 8 weeks after immunization. Mouse spleens and mesenteric lymph nodes (MLN) were obtained and ground through a sterile steel mesh into complete RPMI-1640 medium (Gibco, Auckland, New Zealand). After being centrifuged at 1500× *g* for 10 min, the pellets were obtained, and the spleen and MLN cells were isolated as previously described [32,61], 2 × 10^6^ cells/mL were cultured in RPMI-1640 medium containing 3% fetal bovine serum (FBS), penicillin (100 U/mL) and streptomycin (100 μg/mL). After stimulation with 4 μg/mL rTsT for 72 h at 37 °C, the supernatant was collected and concentration cytokines (IFN-γ and IL-4) were determined using a sandwich ELISA [62,63]. 

### 2.10. Antibody-Dependent Cellular Cytotoxicity (ADCC)

Anti-rTsT antibody dependent cytotoxicity on *T. spiralis* newborn larvae was performed as reported before [64,65]. Briefly, the microtiter plate was used for the ADCC test. Each assay was done in triplicate. A 200-μL volume of RPMI 1640 medium containing 100 larvae and 2 × 10^5^ mouse peritoneal exudate cells (PECs) were added into each well and kept at 37 °C for 24–72 h. The medium was first supplemented with diverse sera (1:50–1:800 dilutions of anti-rTsT immune serum, *T. spiralis* infection serum or pre-immune serum). The serum samples used in this study were not heat-inactivated to inactivate complement. The worm viability following ADCC was judged in accordance with larval morphology and mobility. The living worms were active and exhibited mobile, the killed larvae were straight and inactive [66]. The cytotoxicity results were shown as the percentage of the killed larvae to total number of larvae used in each experiment [67].

### 2.11. Larval Challenge and Evaluation of Protection Efficacy

To assess the protection efficacy produced by immunization with rTsT, the remaining 20 mice of each group was each challenged orally with 300 infectious *T. spiralis* ML at 2 weeks after the last boost. At 5 dpi, 10 mice of each group were euthanized to collect the adult worms in intestines. The muscle larvae were recovered through artificial digestion of muscle tissues of additional ten mice of each group at 30 dpi [68]. The immune protective effect induced by rTsT immunization was assessed as the worm reduction of enteral adult worms and larvae per gram (LPG) of skeletal muscle tissues of immunized mice compared with the worms recovered from the PBS group [69,70].

### 2.12. Histopathological Examination of Muscle and Intestines from Infected Mice

At day 5 or day 30 after challenge, small intestine and masseter muscles were excised from infected mice and fixed in 4% formalin for 24 h and embedded in paraffin, 3-μm-thick sections of muscle tissues were prepared, deparaffinized and stained using hematoxylin and eosin (HE) stain. The sections were examined under microscopy, and the inflammatory cells (eosinophils, neutrophils and lymphocytes) around encapsulated *T. spiralis* larvae on muscle sections from three groups of mice were counted per field (200×) as previously described [30,71].

### 2.13. Statistical Analysis

All the data were statistically compared using SPSS for Windows, version 21.0 (IBM, Chicago, IL, USA). The data were shown as the mean ± standard deviation (SD). The differences of different groups was analyzed by a Chi-square test or one-way ANOVA. The relationship between ADCC cytotoxicity and serum dilution/culture time was compared using correlation analysis *p* < 0.05 was regarded as statistically significant. 

## 3. Results

### 3.1. Bioinformatics Analysis of TsT

**Figure 2:**
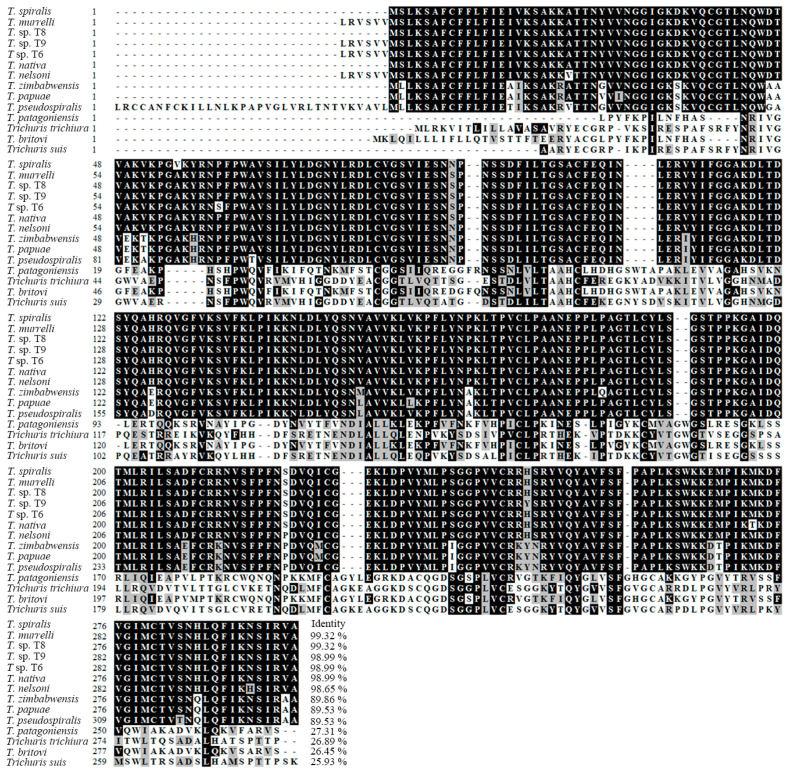
Sequence alignment of *Trichinella spiralis* trypsin gene (GenBank: XP_003374437.1) with other *Trichinella* species/genotypes and *Trichuris suis*. The sequences of trypsin from various organisms were analyzed using Clustal W, the obvious difference was found in diverse *Trichinella* species or genotypes. Black shade represents the same residues with TsT, and grey shade indicates the conservative substitutions.

**Figure 3:**
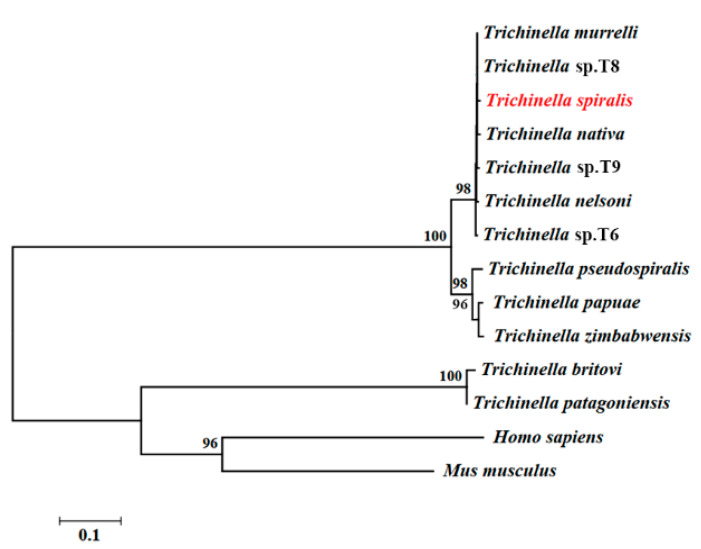
Neighbor-joining phylogenetic tree of trypsin.

Complete TsT cDNA sequences are 891 bp encoding 296 aa, with a molecular weight (MW) of 32.97 kDa and isoelectric point (pI) of 9.42. TsT had a signal peptide at 1-18 aa. A transmembrane prediction using Hidden Markov Model (TMHMM) analysis revealed that TsT has no transmembrane. Subcellular location predicted that TsT might be a secretory protein. The homology comparison of TsT amino acid sequences with trypsin of other *Trichinella* species/genotypes was presented in Figure 2. The amino acid sequences of the TsT had an identity of 99.32, 99.32, 98.99, 98.99, 98.99 and 98.65% with trypsin of the 6 encapsulated species/genotypes of the genus *Trichinella* (*T. murrelli,* T8, T9, T6, *T. nativa*, and *T. nelsoni*), and the TsT had an identity of 89.86, 89.53, and 89.53% of trypsin from three non-encapsulated *Trichinella* species (*T. zimbabwensis*, *T. papuae*, and *T. pseudospiralis*). Phylogenetic analysis of TsT with trypsin of other *Trichinella* species/genotypes was shown in Figure 3. Phylogenetic tree showed that a monophyletic group of the genus *Trichinella* was well supported except the two species of *T. britovi* and *T. patagoniensis*. *T. spiralis* has the more close evolutionary relationship with encapsulated species of the genus based on the phylogenetic analysis of trypsin. 

### 3.2. Expression and Western Blot Analysis of rTsT

**Figure 4:**
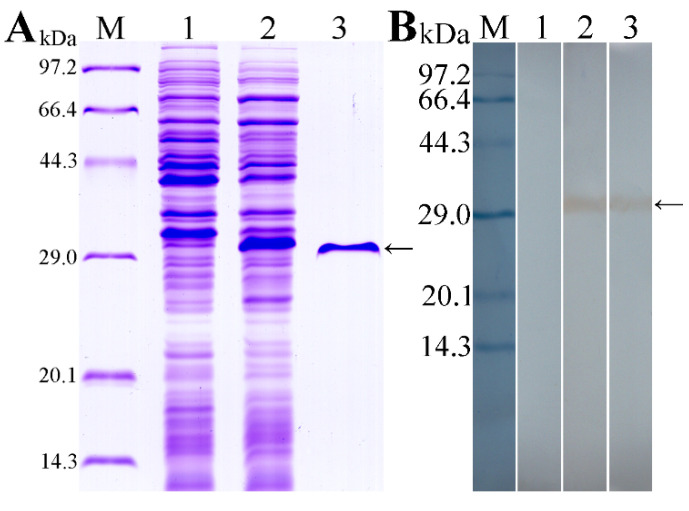
Expression and identification of recombinant TsT protein. (**A**): Expression and purification of rTsT. SDS-PAGE analysis showing rTsT was expressed in IPTG-induced *E. coli* lysates (lane 2), but not expressed in *E. coli* lysate prior to induction (lane 1). Lane 3: the purified rTsT protein. (**B**): Western blot analysis of the rTsT immunoreaction. Lane 1: un-induced *E. coli* lysate was not recognized by anti-his McAb. Specific recognition of expressed rTsT in induced *E. coli* lysate (Lane 2) and purified rTsT by anti-his McAb. Arrow shows the rTsT.

**Figure 5:**
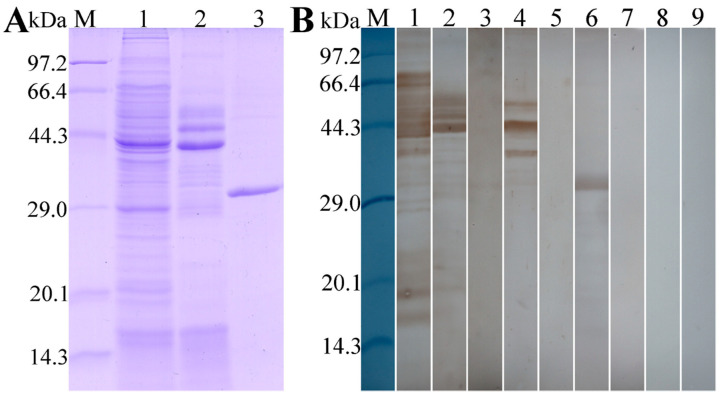
Identification of rTsT antigenicity. (**A**): SDS-PAGE analysis of ML crude antigens (lane 1), ES antigens (lane 2) and purified rTsT (lane 3). (**B**): Identification of rTsT antigenicity on Western blotting. ML crude antigens (lane 1), ES antigens (lane 2) and purified rTsT (lane 3) were probed by mouse infection sera. Natural TsT in ML crude antigens (lane 4) and rTsT (lane 6) were identified by anti-rTsT serum, but no natural TsT in ML ES antigens (lane 5) was probed by anti-rTsT serum. The ML crude (lane 7) and ES antigens (lane 8), and rTsT (lane 9) were not identified using murine pre-immune serum.

As shown in Figure 4A, recombinant BL21 carrying pQE-80L/TsT expressed a 30.9 kDa fusion protein. The purified rTsT presented a clear individual protein band. The MW (30.9 kDa) of rTsT protein was the same as its predicted size. On Western blot analysis, rTsT was identified using anti-his tag monoclonal antibody (McAb) (Figure 4B), infection serum and anti-TsT serum (Figure 5). Several native TsT with 32.9–55.4 kDa in *T. spiralis* ML crude antigens was detected by anti-rTsT serum, but not in ML ES protein (Figure 5), demonstrating that TsT is a worm somatic protein, not a secretion protein of ML stage. 

### 3.3. TsT Transcription in Diverse T. Spiralis Stages

**Figure 6:**
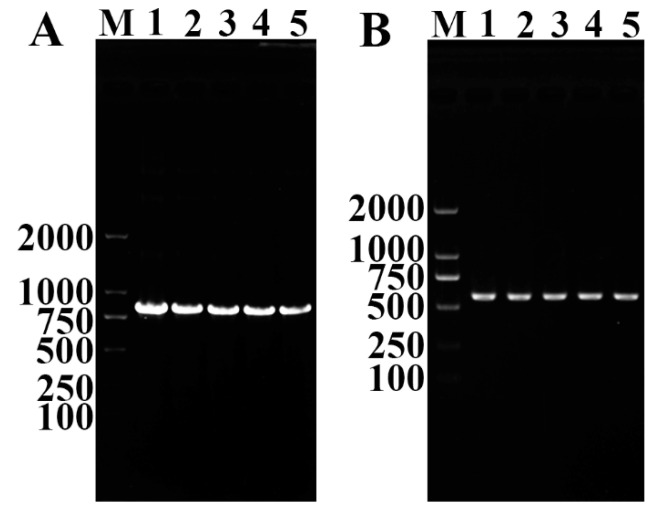
RT-PCR analysis of TsT (**A**) and GAPDH (**B**) mRNA expression at *T. spiralis* muscle larvae (lane 1), intestinal infective larvae (lane 2), 3-day adult worms (lane 3), 6-day adult worms (lane 4), and newborn larvae (lane 5).

As shown in Figure 6A, the TsT with a predicted size of 837 bp was detected by RT-PCR in diverse lifecycle stage worms of *T. spiralis*, including ML, IIL, 3- and 6- day AW, and NBL, the housekeeping gene (GAPDH) was also amplified in various stage worms as a positive control (570 bp) (Figure 6B). 

### 3.4. Expression and Localization of Native TsT in Various T. spiralis Stages

**Figure 7:**
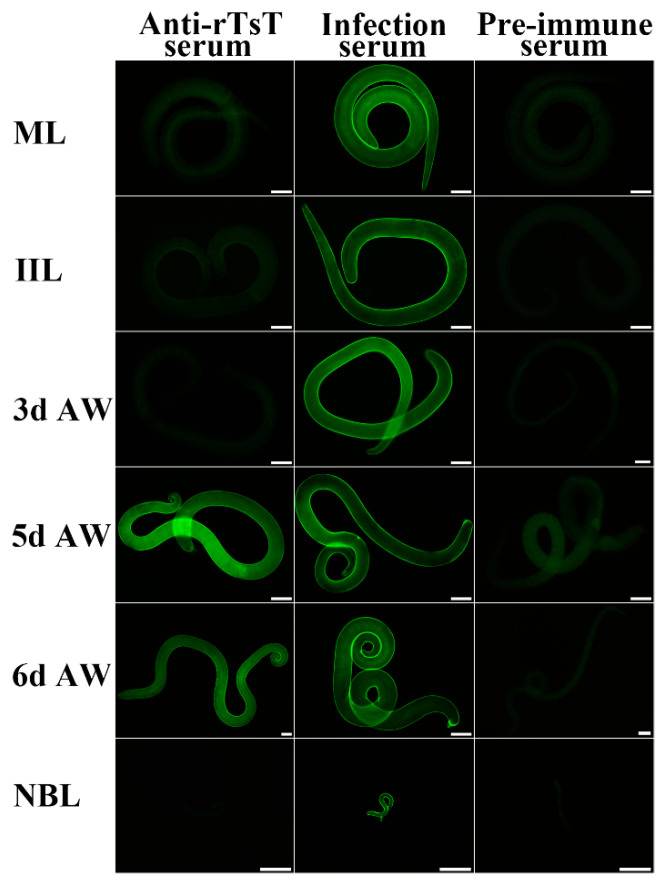
Expression and localization of TsT at the cuticle of diverse *T. spiralis* worm stages by IIFT. The intact whole worms were incubated with anti-rTsT serum, and positive immunofluorescence staining was localized at the epicuticle of only 5- and 6- day adult worms. Pre-immune serum did not identify any cuticle of all lifecycle stages of *T. spiralis*. Bar, 50 μm.

**Figure 8:**
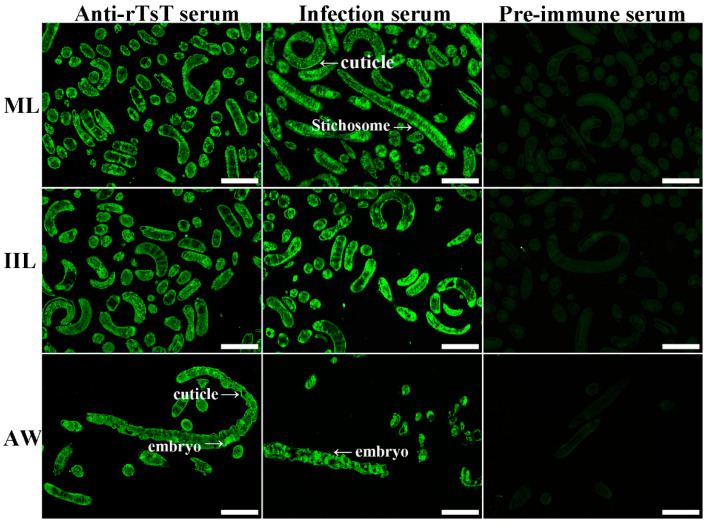
Tissue localization of TsT in worm cross-sections of *T. spiralis* ML, IIL and AW stages by IIFT. Fluorescence was observed at the whole worm section of ML and IIL, and intrauterine embryos of female adult. No immunostaining in worm cross-sections was found using pre-immune serum. Bar, 100 μm.

The results of IIFT with the whole worms showed that when anti-rTsT serum was used, the fluorescence immunostaining was observed on the epicuticle of only 5 and 6 day adults, not at ML, IIL, 3 day AW and NBL (Figure 7). After incubation with anti-rTsT serum, the immunostaining was localized at the whole worm section of ML and IIL, and intrauterine embryos of the adult females (Figure 8). No worm components were identified using pre-immune serum.

### 3.5. Anti-TsT Antibody Response

**Figure 9:**
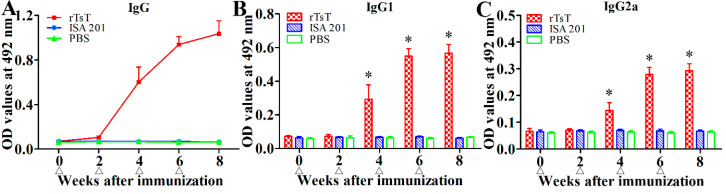
Humoral immune responses against rTsT in immunized mice measured by ELISA. (**A**) Serum level of total anti-rTsT IgG in immunized mice, and adjuvant or PBS control mice. The IgG1 (**B**) and IgG2a (**C**) subclass responses against rTsT were detected at different time points following vaccination. The data are presented as mean ± SD for 20 mice per group. The vaccination time points are marked using triangle (∆). * Significantly different with respect to the ISA 201 or PBS control group (* *p* < 0.0001).

To determine anti-TsT antibody response, rTsT-specific IgG and IgG1/IgG2a in sera of all vaccinated mice were measured by rTsT-ELISA. Anti-rTsT IgG level was notably increased following the second immunization, anti-rTsT IgG titers reached 1: 10^5^ two weeks after last immunization, suggesting that the rTsT is strong immunogenic. Furthermore, no mice injected with adjuvant ISA 201 or PBS alone showed any anti-rTsT IgG response (Figure 9A). The IgG1 level on weeks 4, 6 and 8 after immunization was obviously higher than IgG2a (*t*_4w_ = 7.327, *t*_6w_ = 5.916, *t*_8w_ = 20.751, *p* < 0.0001) (Figure 9B,C), indicating that immunization with rTsT elicited the Th2-predominant concomitant Th1/Th2 response. 

### 3.6. Enteral Mucosal Immune Response Induced by rTsT Immunization

**Figure 10:**
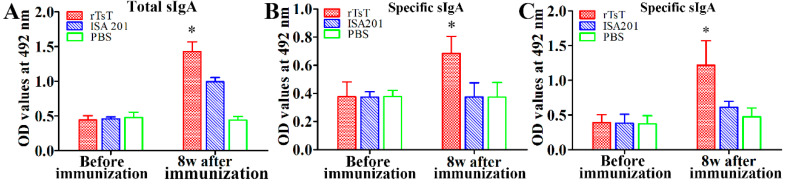
Levels of total sIgA (**A**), anti-TsT specific sIgA in enteral fluid of vaccinated mice assayed by ELISA using ML crude antigens (**B**) and rTsT (**C**). The data are the mean ± SD for 5 mice per group. * Significantly different compared with the ISA 201 or PBS group (* *p* < 0.01).

To evaluate enteral mucosal sIgA response to rTsT immunization, total sIgA and TsT-specific sIgA were assayed by ELISA. The results showed that the levels of total sIgA and TsT-specific sIgA of three groups had no significant difference before immunization (*p* > 0.05). Total sIgA level in mice immunized with rTsT at 8 weeks following immunization was clearly higher than those of ISA 201-injected mice and PBS group (*F* = 137.180, *p* < 0.0001) (Figure 10A). When The ELISA with ML crude antigens and rTsT were used, the anti-TsT sIgA levels in immunized mice were significantly higher than the mice injected with only ISA 201 or PBS (*F*_ML_ = 13.567, *F*_rTsT_ = 16.042; *p* < 0.01) (Figure 10B,C). Enteral specific sIgA was not detected in mice injected with only adjuvant or PBS. The results suggested that subcutaneous vaccination with rTsT produced not only systemic antibody response but also significant intestinal local mucosal sIgA response.

### 3.7. Cytokine Responses to the rTsT Immunization 

**Figure 11:**
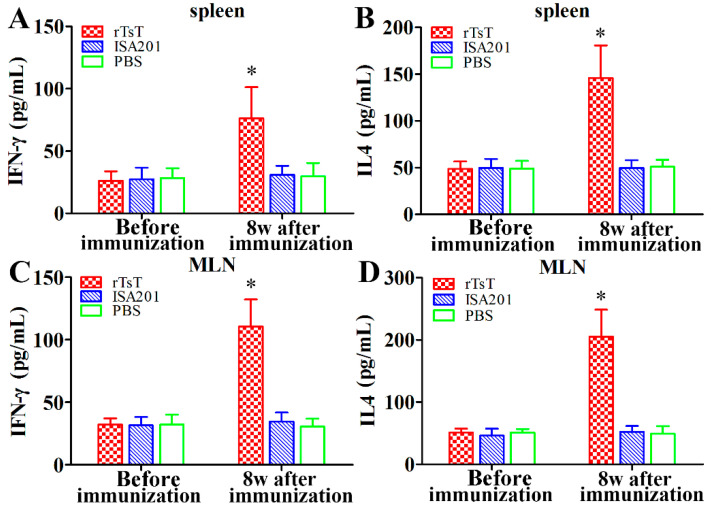
Cytokines secreted by spleen (**A**,**B**) and MLN (**C**,**D**) cells on rTsT stimulation. The assay of the cytokine IFN-γ and IL-4 was performed on the cells stimulated for 72 h with rTsT. The data are the mean ± SD of cytokine levels for 5 mice per group. * Significantly different with respect to the ISA 201 or PBS control group (* *p* < 0.01).

The ELISA results revealed that level of IFN-γ and IL-4 among three groups had no significant difference prior to immunization (*p* > 0.05). But the level of the two cytokines in rTsT-immunized mice 8 weeks after immunization were significantly higher than the ISA 201 and PBS group (*p* < 0.01) (Figure 11). Our results demonstrated that immunization with rTsT elicited the concomitant Th1/Th2 responses, indicating that subcutaneous immunization with rTsT induced both the systemic (spleen) and local enteral mucosal (MLN) cellular immune response.

### 3.8. Killing and Destruction of the NBL by PECs and Anti-rTsT Immune Serum

**Figure 12:**
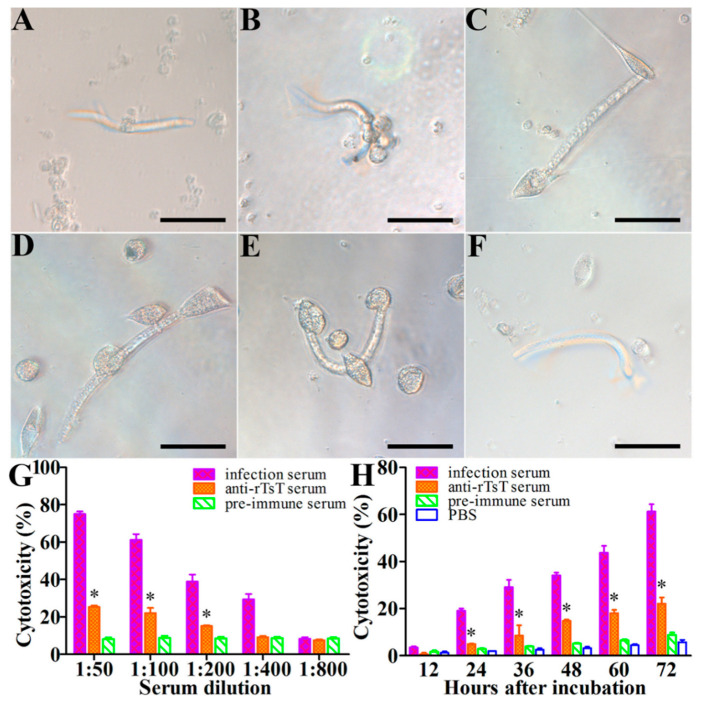
Cytotoxic activity of PECs on the NBL in the presence of anti-rTsT immune serum. (**A–D**): Morphology of *T. spiralis* newborn larvae co-incubated with the PECs and anti-rTsT immune serum for 24 h (A), 48 h (B), 60 h (C) and 72 h (D). (**E**) *T. spiralis*-infected murine sera as positive control. (**F**): Pre-immune sera as negative control. Bar, 100 μm. (**G**): The cytotoxic activity of anti-rTsT immune serum was antibody dose-dependent. (**H**): The cytotoxic activity of anti-rTsT immune serum was also related to the incubation time. * Significantly different with respect to the pre-immune serum group (* *p* < 0.01).

The cytotoxic activity of anti-rTsT immune serum on the NBL was assessed by the ADCC test. After cultivation, anti-rTsT immune serum promoted the PECs adhesion and NBL destruction in the presence of anti-rTsT immune serum (Figure 12). Comparison of the cytotoxic capacity of various sera diluted 1:100 revealed that the percent of the dead NBL in anti-rTsT immune serum group (21.97%) was statistically higher than the pre-immune serum group (8.77%) (χ^2^ = 6.733, *p* < 0.01). The cytotoxic activity of anti-rTsT immune serum was dose-dependent of anti-rTsT antibodies (*r* = 0.880, *p* < 0.001), and exhibited a declining trend with the immune serum dilution increase (*F =* 101.254, *p* < 0.001). The cytotoxic activity of anti-rTsT immune serum was also related to the incubation time (*r* = 0.993, *p* < 0.001) and revealed an increasing mortality of the NBL with extension of incubation time (*F* = 88.941, *p* < 0.001) (Figure 12). 

### 3.9. Immune Protection Induced by rTsT Immunization 

**Figure 13:**
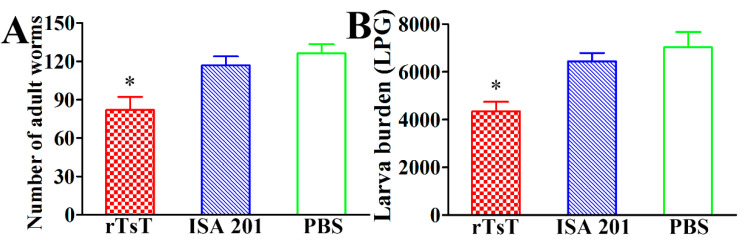
Immune protection induced by rTsT immunization after challenge with 300 *T. spiralis* larvae. (**A**): Intestinal AW burden at 5 days after challenge; (**B**): Muscle larval burden (larvae per gram of skeletal muscle tissue, LPG) at 30 days after challenge. The parasite burdens are shown as the mean ± SD for 10 mice per group. * *p* < 0.01 compared to the ISA 201 and PBS groups.

Compared to the PBS group, vaccination of mice with rTsT showed a 33.17% enteral AW reduction at 5 dpi (Figure 13A) and a 37.80% ML reduction at 30 dpi (Figure 13B) following challenge with 300 *T. spiralis* infectious larvae (*F*_AW_ = 79.988, *F*_ML_ = 8.683, *p* < 0.01). The results indicated that immunization of mice with rTsT induced a partial immune protection against *T. spiralis* challenge.

### 3.10. Intestinal and Muscle Histopathological Change of Infected Mice

**Figure 14:**
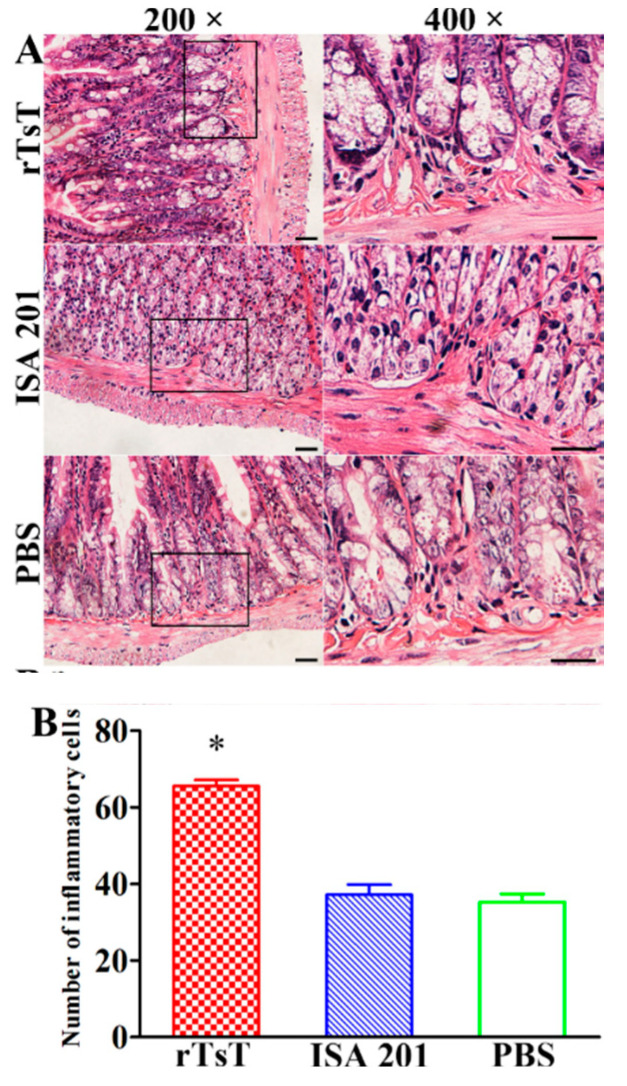
Intestinal histopathological change of infected mice at 5 days after challenge. The intestinal sections were stained using hematoxylin and eosin (HE), and examined under microscopy. (**A**): Inflammatory cell infiltration of intestinal mucosa in three groups of infected mice. (**B**): Quantification of intestinal mucosal inflammatory cells on intestinal sections. * *p* < 0.001 compared to the adjuvant and PBS groups. Scale-bars: 100 μm.

**Figure 15:**
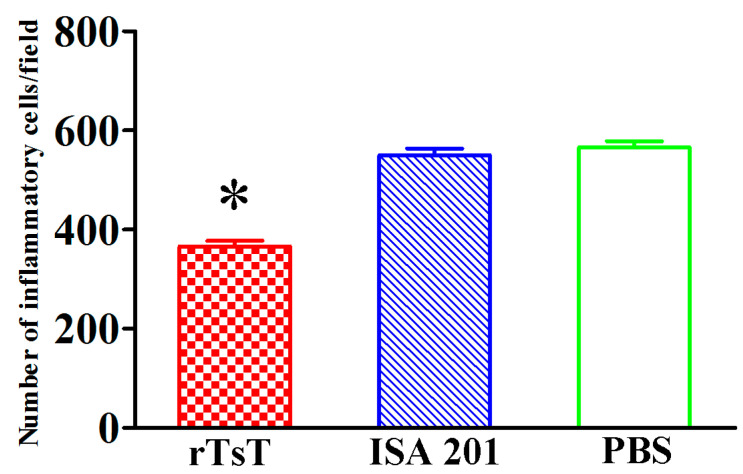
Muscle histopathological change of infected mice at 30 days after challenge. The masseter muscle sections were stained using hematoxylin and eosin (HE), and examined under microscopy. The inflammatory cells around encapsulated *T. spiralis* larvae on muscle sections from three groups of mice were counted per field (200×). * *p* < 0.001 compared to the adjuvant and PBS groups. Scale-bars: 100 μm.

Histopathological changes of the intestines and masseter muscles of different groups of mice was examined at days 5 and 30 following challenge. The results showed that the number of inflammatory cells of intestinal mucosa in rTsT-immunized mice was obviously higher than the adjuvant and PBS control group (*F* = 258.842, *p* < 0.001) (Figure 14), whereas the number of inflammatory cells around encapsulated larvae in skeletal muscles in rTsT-immunized mice was obviously reduced compared to the adjuvant and PBS group (*F* = 199.256, *p* < 0.001) (Figure 15). The results suggesting that rTsT immunization appeared to enhance intestinal inflammatory infiltration in immunized mice which may accelerate the expulsion of adult worms from the gut. Furthermore, rTsT immunization alleviate the inflammation infiltration and relieve the *Trichinella* infection in skeletal muscle tissues of immunized mice.

## 4. Discussion

Serine proteases have two main functional domains, trypsin-like domains and subtilisin-like domains. Most trypsin-like domains exert a major act in parasite invasion, digestion and degradation of host’s protein components and immune evasion [72,73]. A *T. spiralis* adult serine protease gene Ts-ADSp-7 has a trypsin-like serine protease domain without substrate binding sites and protease activity, but has the capacity to inhibit host’s blood coagulation [74]. The silencing of *T. spiralis* serine protease 1.2 (TsSP1.2) by siRNA mediated RNA interference suppressed larval invasion and development [53]. Immunization of mice with another *T. spiralis* serine protease Ts31 exhibited a 53.5% ML reduction of muscle tissues following challenge [52]. A chymotrypsin-like enzyme of *T. spiralis* (Tschy) promoted larval penetration into enteral epithelia [54]. These results suggeted that the serine proteases and trypsin participated in larval invasion and development, they might be the candidate targets for anti-*Trichinella* vaccines. However, studies on *T. spiralis* trypsin have not been reported in the literatures to the present.

In this study, the TsT gene was cloned and expressed in *E. coli* expression system. Sequence analysis on the TsT gene showed TsT had an identity of 99.32, 99.32, 98.99, 98.99, 98.99 and 98.65% with trypsin of the 6 encapsulated *Trichinella* species/genotypes (*T. murrelli,* T8, T9, T6, *T. nativa*, and *T. nelsoni*). Phylogenetic tree showed that a monophyletic group of the 7 encapsulated *Trichinella* species was well supported except *T. britovi* and *T. patagoniensis*. The trypsin enzymatic activity of rTsT was not detected on gelatin zymography in this study. The absence of enzymatic activity of rTsT might be owing to the incorrect folding of rTsT in *E. coli* [52]. Therefore, it is necessary to utilize the eukaryotic expression system to prepare rTsT with enzymatic activity. After purification, the rTsT was applied to produce anti-rTsT immune serum. Vaccination of mice with rTsT produced the high level of serum anti-rTsT IgG response, anti-rTsT IgG titer of immune sera was 1: 100,000, indicating that rTsT had a good immunogenicity. 

Western blotting results revealed that rTsT was recognized by anti-rTsT serum and infection serum. Several natural TsT with 32.9–55.4 kDa in ML crude protein were identified with anti-rTsT serum, but not in ML ES protein (as shown in Figure 5), suggesting that TsT is a worm somatic protein, not a secretory protein of ML stage. The recognition of several native TsT in ML crude proteins might be because TsT have diverse isoforms, or this TsT protein could be processed by post-translational modification and processing. It is also likely because the TsT is a member of *T. spiralis* serine protease family which possessed the same antigenic epitopes [20,44,47,49]. RT-PCR results revealed that TsT gene was transcribed at all of *T. spiralis* lifecycle stages (e.g., ML, IIL, 3 and 6 day AW, and NBL). The IIFT result showed that natural TsT was localized at whole section of ML and IIL, and principally at epicuticle of 5–6 day adults, suggesting that the TsT is a somatic antigen of this paraiste and it is adult-stage specific surface antigen. *Trichinella spiralis* surface antigens are composed of the epicuticle proteins themselves and ES proteins which were incorporated on the epicuticle [75]. They are directly contacted with the host immune system, are the major target antigens which elicit the immune responses, and might play a significant effect in *T. spiralis* worm development and immune evasion in the host [21]. 

To investigate the humoral and cellular immune responses triggered with rTsT immunization, the levels of antibody and cytokines elicited by immunization with rTsT was measured in this study. Our results showed that immunization using rTsT produced distinctly elevation of anti-TsT antibodies (serum IgG, IgG1/IgG2a, and intestinal sIgA), and the rTsT immunization also induced both of systemic (spleen) and local intestinal mucosal (MLN) cellular immune response, demonstrated by a clear rise of cytokines (IFN-γ and IL-4). To investigate which are the main cells producing IFN-γ and IL4, different immune cell populations (CD4^+^ IFN-γ^+^/CD4^+^ IL-4^+^ cells) in spleen and MLNs of immunized mice need to be determined by flow cytometry analysis in future studies [70]. Furthermore, it is necessary to quantify the infiltration of dendritic cells, macrophages, monocytes, T and B cells in spleen of immunized mice in further experiment. The mixed Th1/Th2 response exerted a crucial act for protective immunity to *T. spiralis* infection [32,76,77]. Specific anti-*Trichinella* IgG also took part in the rapid worm discharge of *T. spiralis* adults from the guts [78]. Additionally, the rTsT immunization appeared to strengthen the enteral inflammatory infiltration in immunized mice, which may accelerate the AW expulsion from the gut. Previous studies indicated that anti-*Trichinella* sIgA participated and accelerated the parasite dislodgement from the intestine, passive transfer of mice with anti-*Trichinella* IgA exhibited an overt immune protective effect against challenge infection [79]. Intestinal sIgA also suppressed the female adult reproductive capacity of *T. spiralis* [62]. To assess the cytotoxic activity of anti-TsT immune serum, the ADCC test was conducted in this study. The results showed that anti-rTsT immune serum promoted the adhering and killing of the PECs to the NBL, and the cytotoxic activity was dose-dependent of anti-rTsT antibodies, suggesting that anti-TsT IgG participated in the killing and destruction of newborn larvae by the ADCC fashion [56,64]. The sera used in this study were not heat-inactivated to inactivate complement. In previous ADCC tests, the newborn larvae incubated with immune sera without PECs were not destroyed, indicating that only immune sera without PECs does not induce the cytotoxicity [28,80]. Furthermore, IFN-γ also acts an important protection against *T. spiralis* newborn larvae [81].

Following challenge with 300 *T. spiralis* infectious larvae, the mice immunized with rTsT showed a 33.17% reduction of enteral AW at 5 dpi and a 37.80% ML reduction at 30 dpi, respectively. The worm burden reduction observed in this study is similar to those obtained with subcutaneous immunization using other individual recombinant *T. spiralis* protein [19,28,52], but the protective level obtained in this study is lower than those of previous reports by using oral or intranasal vaccination [76,82]. The immune protection obtained in this study maybe owing to the results of a combination of worm expulsion from the gut, reduction of female fecundity, or ADCC killing and destruction of NBL induced by rTsT immunization, which resulted in a reduction of intestinal adults and muscle larvae in immunized mice. Our results demonstrated immunization of mice with a single *T. spiralis* protein (TsT) only produced a partial immune protective effect against larval challenge, the infective larvae in muscle tissues of vaccinated animals were not fully eradicated. *Trichinella spiralis* is a multicellular foodborne parasite with a complicated lifecycle, each developmental phase has the stage-specific antigens [83,84]. Hence, to eliminate *Trichinella* infection in food animals, it is needed to develop the oral polyvalent anti-*Trichinella* vaccines which should be comprised of multiple antigenic epitopes of various *T. spiralis* lifecycle phases [57,70]. 

## 5. Conclusions

In conclusion, TsT was a *T. spiralis* somatic antigen and adult-stage specific surface antigen, and it had a good antigenicity. Vaccination of mice with TsT induced a systemic mixed Th1/Th2 response and an intestinal local sIgA response, which produced a partial protection against *T. spiralis* larval challenge. The results showed that TsT plays a role in *T. spiralis* development and survival in host, and it might be considered as a potential target antigen for anti-*T. spiralis* vaccines. However, to improve its protective efficacy, oral anti-*Trichinella* vaccines comprised of multiple antigenic epitopes of various *T. spiralis* lifecycle phases should be developed in further study.

## 6. Ethics Approval and Consent to Participate

The experimental animals are raised and cared on the basis of the National Guidelines for Experimental Animal Welfare of the People’s Republic of China (2006). The study was approved by approval was acquired from the Life Science Ethics Committee of Zhengzhou University (No. SCXK 2017–0001).
